# Artificial Urinary Sphincter Complications: A Narrative Review

**DOI:** 10.3390/jcm13071913

**Published:** 2024-03-26

**Authors:** Ryan L. Frazier, Marilyn E. Jones, Matthias D. Hofer

**Affiliations:** 1 San Antonio Uniformed Services Health Education Consortium, Brooke Army Medical Center, 3551 Roger Brooke Drive, Fort Sam Houston, TX 78234, USA; ryanlfrazier3@gmail.com (R.L.F.); marilynellise@gmail.com (M.E.J.); 2 Urology San Antonio, 18915 Meisner Drive, San Antonio, TX 78258, USA

**Keywords:** artificial urinary sphincter, complications, prosthesis

## Abstract

Stress urinary incontinence is a financially burdensome and socially isolating problem and can be experienced by men as a result of radical prostatectomy, radiation therapy, or other urologic surgery. Artificial urinary sphincter (AUS) placement for stress urinary incontinence is considered the ‘gold standard’ for male stress urinary incontinence. While initially only placed by specialized prosthetic surgeons, changes in urologic training have made implantation of the device by general urologists more widespread. Additionally, even though a minority of urologists place the majority of implants, many urologists may find themselves caring for patients with these devices even if they have never placed them themselves. For this reason, it is paramount that the urologic surgeon implanting the device and those caring for patients with prostheses are familiar with the various perioperative and postoperative complications of AUS implantation. This review discusses the most commonly reported complications of AUS implantation as well as those that are rarely described. Knowledge of these potential complications is necessary in order to care for patients with urologic implants.

## 1. Introduction

Urinary incontinence in men is associated with high financial burden, decreased quality of life, and poor health outcomes. The estimated prevalence of urinary incontinence in men varies from 11% to 31% and increases with age [1]. In contrast to women, stress urinary incontinence occurs less frequently in men compared to urge urinary incontinence. The etiology of stress urinary incontinence in males is most associated with prior urologic surgery, trauma, or urethral stricture disease. Management options for stress urinary incontinence in men range from conservative measures like patient directed pelvic floor exercises and pelvic floor physical therapy with biofeedback to surgical prosthetic implantation.

The gold standard for the treatment of severe stress urinary incontinence in men is artificial urinary sphincter (AUS) implantation. The device was first described in 1947 by Trost and Elliot and the first successful implantation occurred in 1972 at the Baylor College of Medicine. There were several iterations of the device until the development of the Boston Scientific AMS 800^TM^, which was released in 1981. Since the time of its release, there have been few improvements to the device, with the most recent in 1987 [2]. Despite this, it remains the most widely used device on the market. The AMS 800^TM^ is a three-piece device composed of a circumferential periurethral cuff, which provides coaptation of the patient’s urethra; a pressure regulating balloon, which regulates the appropriate pressure within the system; and a button, which allows the patient to void volitionally and healthcare providers to control the prosthetic for urethral instrumentation. Other AUS models that have been certified for implantation include the AUS Flow Secure which is a one-piece device developed in 2006 by Sphinx Medical in the United Kingdom and the Zephyr ZSI375 AUS, Zephyr Surgical Implants, Geneva, Switzerland. This is another one-piece device developed by Zephyr Surgical Implants in Switzerland [3]. This review will focus on data regarding the AMS 800^TM^ as the majority of the published research pertains to this model and it is the most widely used device for the treatment of urinary incontinence.

The artificial urinary sphincter has been shown to achieve short- and long-term continence rates and to improve quality of life of patients [4,5]. The MASTER trial compared the outcomes of AUS implantation and male sling placement and demonstrated non-inferiority between the two devices. The male urethral sling is a device which uses a piece of mesh to provide greater sphincteric coaptation and historically has been used for less severe cases of stress urinary incontinence in men. The trial demonstrated an 84% continence rate following AUS placement. Of the men in the study, 90.6% reported that they were completely or fairly satisfied with their continence and 84.5% of men reported that they would recommend AUS implantation to a friend with similar symptoms. These data demonstrate that patient satisfaction following AUS implantation is high despite not achieving complete continence [6].

Prosthetic implantation is not without risk, despite the above noted positive outcomes. Risk factors for poor surgical outcome have been well-studied. As in non-prosthetic surgery, previously radiated and reoperative surgical fields increase the risk of complication. This includes the category of ‘high risk urethras’, which have undergone urethral reconstruction, radiation, or some other form of vascular compromise [7,8]. Similarly, patients on anticoagulation or with coagulopathy have increased risk of bleeding and hematoma formation [9]. All surgeons are aware of the morbidity and mortality associated with synthetic material infection. Complications requiring explantation have been associated with a patient medical history of diabetes, hypertension and cardiovascular disease, low albumin, medical frailty, smoking status, and low serum testosterone [9,10,11,12,13,14,15]. However, infection alone is not the only potential complication which may arise from AUS implantation. It is incumbent upon all prosthetic urologists to be aware of the less common complications, their presentations, and potential management strategies to improve patient outcomes. This narrative review will discuss peri- and postoperative complications of artificial urinary sphincter implantation as reported in the literature.

## 2. Complications of Artificial Urinary Sphincter Surgery

### 2.1. Perioperative Complications

Complications related to AUS implantation can be divided into two major categories: perioperative and late postoperative. In the literature review performed by these authors, there was no consensus regarding a strict time division between these two categories, with some authors reporting perioperative complications as far out as 90 days [16]. Perioperative complications range in severity from mild to devastating. Similarly, complications like scrotal hematomas can be directly related to intraoperative findings and technique, while others, like device fluid loss may be due intrinsically to the device itself. Complications such as respiratory depression requiring re-intubation, renal failure, cerebrovascular accident, or cardiac arrest are catastrophic events that can generally be attributed to complications arising from anesthesia or pre-existing patient comorbidities.

#### 2.1.1. Hematoma

One perioperative complication that may occur with any scrotal surgery and is commonly described following AUS placement is the development of a scrotal hematoma. They tend to occur 1–3 days post-operatively and may be attributed to inadequate hemostasis prior to incision closure, premature reinitiation of anticoagulation, or inadequate scrotal support in the post-operative period among other causes. In a case series of 84 patients, perioperative anticoagulation was found to be an independent risk factor for explantation within the first 12 months postoperatively. In total, 9 of 31 patients undergoing anticoagulation therapy (30%) required explanation as compared to 3 of 53 patients not undergoing anticoagulation (6%). The authors posit poor microcirculation as a result of cardiovascular comorbidities that necessitate anticoagulation as their primary hypothesis [10]. It stands to reason that hematoma formation due to reinitiation of anticoagulation may also be a related cause. While generally a mild complication, hematomas may result in increased pain, poor cosmetic outcome, and contribute to pump migration. In more severe cases, large or expansile scrotal hematomas may become infected which can put the device at risk for infection or erosion. In some instances, especially if of large volume, the hematoma should be evacuated by surgery in order to prevent the above noted sequelae, device migration, or device failure. While this means that the patient needs to undergo another surgery, the evacuation is usually very quick.

#### 2.1.2. Pump Migration

Pump migration, another common complication, is movement of the pump from its original location of placement. In one of the largest case series in the literature, of 404 patients undergoing revision surgery, 11% (*n* = 44) were due to either pump malposition, tube kinking, or a similar issue [17]. While not necessarily a complication inherently, it can cause problems related to patient use either due to discomfort or device function. Patients may present primarily in the office with the complaint of inability to use their device or, in more extreme cases, in retention or renal failure due to chronic inability to empty their bladder. Described problems that have arisen from migration of the pump are poor ease of access, adhesion to intrascrotal structures, and ultimately the need for reoperation for relocation of the component [18,19]. Typically, the pump migrates to an alternative location within the scrotum not necessarily intended by the surgeon. Relocation is a straightforward and expedient procedure. However, rare cases of device migration out of the scrotum and into the abdomen have been described in case reports [20]. If migration of any device component is suspected, plain film X-ray or fluoroscopy can be useful adjunctive tools in addition to physical exam for confirming device position.

#### 2.1.3. Fluid Loss

Another perioperative complication is continence failure due to device fluid loss. One retrospective study reported that 83% of device revisions conducted for mechanical failure were attributed to fluid loss [21]. Fluid loss is the result of damage to the tubing at time of surgery or technical error in connecting system components. System failure secondary to fluid loss can be identified at time of device activation or in the late postoperative period as discussed below. Findings associated with fluid loss are inability to cycle the device and persistent urinary incontinence following device activation. This is a direct result of poor coaptation of the device on bulbar corpus spongiosum tissue. This complication can be identified early if the fluid loss is severe enough to be noticed by the operative surgeon at the time of intraoperative device cycling. Surgeons may notice similar signs as noted above, as well as pooling of device fluid in their operative field. Surgeons who use radioopaque fluid within their systems may be able to identify this with intraoperative or postoperative plain films, though this is not commonly performed [22,23]. The ultimate solution to system failure due to fluid loss is identification and resolution of the faulty connection or replacement of the responsible component.

#### 2.1.4. Urinary Tract Infection

Urinary tract infection (UTI), hematuria, and urinary retention have all been reported as perioperative complications of AUS implantation [16,24,25,26]. Violation of the urethra or instrumentation of the urethra with either a catheter or cystoscope place the patient at risk. Typically, patients present in the first three to five days postoperatively complaining of dysuria, hematuria, or even retention as an underlying symptom or cause of their urinary tract infection. Urinary tract infection following AUS implantation requires treatment, preferably with culture-specific antibiotics. It does not require any surgical intervention. A retrospective cohort study published in 2020 that analyzed data of 105 patients who underwent primary implantation for non-neurogenic stress urinary incontinence reported that UTI occurred in 7.6% of patients. Furthermore, they reported that UTI was an independent risk factor for device explantation [25]. This highlights the importance of prevention, recognition, and treatment of post-operative urinary tract infection. Hematuria in the post-operative setting should not be dismissed as it may be the initial presenting symptom of several significant complications. Hematuria may occur in the setting of UTI. Of more consequence, it could be an indication of unrecognized urethral injury or device erosion. Patients presenting with hematuria may require further evaluation with cystourethroscopy to rule out the latter complications.

#### 2.1.5. Urinary Retention

Urinary retention occurs postoperatively in 8–32% of patients who undergo AUS implantation, with those who undergo transcorporal cuff placement at a significantly higher risk [27,28]. This can be because of postoperative edema and inflammation due to manipulation of the urethra and corpus spongiosum. Alternatively, patients may present in urinary retention if the device is unintentionally activated following implantation instead of being locked in the open position. Urinary retention in the short-term can be managed with a small (10–14 Fr) urethral catheter. Prosthetic surgeons typically try to avoid prolonged urethral catheterization postoperatively as it can increase the risk of urethral erosion. If retention is persistent, urinary diversion with suprapubic tube is generally recommended for at least one month to give the tissue adequate time to heal. If urinary retention has not resolved at that time, an inappropriately sized cuff may be the cause of retention and will ultimately require surgical revision [24].

#### 2.1.6. Other Surgical Complications

Literature discussing general surgical complications are sparse in the review performed by these authors. Data representing the rate of readmission, reintubation, and outcomes resulting in severe end organ damage, like cardiac arrest, cerebrovascular accidents, or renal failure are not well-represented. One retrospective analysis of complications associated with AUS implantation as compared to those undergoing AUS explantation found increased patient frailty was associated with major complications [12]. These data are in concert with existing literature that suggest increased age and patient frailty results in worsened rates of postoperative mortality, complications, and prolonged length of hospitalization [29]. Evaluating patient frailty may assist in preventing major surgical complications.

Similarly, severe complications can occur intraoperatively and, if recognized, can prevent further morbidity and mortality. If unrecognized, injury to bowel, bladder, or other major structures, like major vasculature, can have dire consequences for patients. These injuries most often occur during pressure regulating balloon (PRB) placement. The pressure regulating balloon can be placed in a variety of locations including the prevesical space and both the high and low submuscular spaces [30,31]. Other prosthetic reservoirs have been placed in the subcutaneous space and there is a case series of PRBs placed in the lateral retroperitoneum [32,33]. The dissection completed for prevesical placement of the PRB is performed blindly using solely anatomic landmarks and tactile sensation. For this reason, surgeon expertise plays a role in both the technical placement and decision-making regarding correct PRB location. In patients who have undergone prior major pelvic surgery and radiation, this dissection can be challenging and places intraabdominal structures at greater risk. Patients with injury to abdominal viscera often present with fevers, malaise, and abdominal pain, as can be expected in the setting of an intraabdominal source of infection.

### 2.2. Late Post-Operative Complications

While the AUS is the gold standard of stress urinary incontinence treatment in men, it is well known that the longevity of the device is a topic of concern among the prosthetic urology community [4]. The largest case series conducted reporting device longevity were from a single, high-volume institution. These case series, first reported by Lindner and colleagues in 2015, reported device reoperation rates and follow-up to a median of four years. They found surgery-free survival rates at 1, 5, 10, and 15 years to be 90%, 74%, 57%, and 41%, respectively [34]. These data were updated in 2020 by Boswell and colleagues who provided further analysis in a larger patient cohort with greater long-term follow-up to a median of almost five and a half years. The authors’ updated case series observed surgery-free survival rates at 5, 10, 15, and 20 years to be 72%, 56%, 41%, and 33%, respectively [17].

Rigorous preoperative counseling with patients is warranted regarding the potential need for reoperation. The most reported and well-studied complications occur in the late postoperative period: device infection, urethral erosion, urethral atrophy, and mechanical malfunction.

#### 2.2.1. Device Infection

Infection of the AUS or exposure of any of the components is a potentially devastating complication and has driven significant changes within the field of prosthetic urology. One needs only to look at the creation of proprietary technology like the InhibiZone Treatment™, which coats the device with a combination of antibiotics, specifically minocycline and rifampin, or recent studies focusing on the use of perioperative antibiotics during prosthetic implantation, to see the value prosthetic surgeons place on infection prevention [35,36,37,38,39,40]. Not only is device infection rife with physical morbidity, it is financially burdensome, necessitating multiple staged procedures often with worsened outcomes [41]. Device infection bridges the gap between perioperative and postoperative complications, as they can occur in both periods. The rate of device infection varies based on the reported series ranging from 1% up to 33.3% depending on approach, with variability noted between the penoscrotal versus perineal approach, single versus tandem cuff placement, and transcorporal placement [7,16,24,42,43,44].

When discussing the difference in outcomes between the perineal and penoscrotal approaches, it is important to acknowledge how they differ technically. Most commonly, AUS placement is performed through a perineal approach; the patient is positioned in dorsal lithotomy and a vertical midline incision is made in the perineum giving direct access to the bulbar urethra. This approach traditionally requires a second incision, most commonly subinguinal, for placement of the pump and pressure regulating balloon. The penoscrotal approach, first described in 2003 by Wilson et al., utilizes an upper transverse scrotal incision which allows for placement of both the pump and PRB through a single incision [45]. In this approach, the patient may be in the supine position. The proximal corpora are exposed allowing for mobilization of the bulbar urethra. Advantages of this technique include a single incision, shorter operative time, and the capability to concurrently implant an inflatable penile prosthesis [10]. Despite these reported advantages, the American Urological Association (AUA) recommends a single cuff, perineal approach citing comparative studies which demonstrate inferior outcomes with the penoscrotal approach [10,46]. Data directly comparing the two approaches are sparse and rely entirely on retrospective analysis. A study published in 2021 directly comparing the two approaches reported that continence outcomes between the two groups were equivocal and that while the perineal approach did have a higher rate of revision or replacement, there was no significant difference in rates of infection, erosion, or mechanical failure [47]. Another retrospective analysis directly comparing the two approaches found that while the perineal approach had a higher rate of complications overall, 44% compared to 26.3%, there was no statistically significant difference in rates of infection when comparing the two approaches [42]. Further study with prospective trials will be required to determine if there is a clinically meaningful difference between the surgical approaches as currently there are too many confounding variables to make a definitive comparison. Similarly, there is no consensus as to the reason for delayed infection. There are some who hypothesize that most late infections are in fact the result of urethral erosion of leading to infection as the predominant presentation [48].

Patients with device infection have varied presentations ranging from dysuria to shock secondary to septicemia. Other common findings and presentations are pain and erythema overlying components of the device, extrusion of device components, a fixed or nonmobile button, hematuria, and urinary tract infection. Infection or extrusion of one or any of the components warrants surgical removal of the device [39]. In select populations with device infections or erosion, salvage device implantation can be performed. Although first popularized by Mulcahy for inflatable penile prosthesis (IPP) infections, the same principles hold true. In this procedure, all components of the infected device, including debris and old suture, are removed. Then, copious irrigation with antiseptic irrigation is performed. The original solution described by Mulcahy and colleagues contained a mixture of vancomycin and gentamicin, half-strength hydrogen peroxide, and half-strength povidone-iodine. The irrigation protocol has changed since its original description in the 1990s. He now recommends pressure irrigation with vancomycin/piperacillin-tazobactam/amphotericin B due to the change in identified pathogenic organisms found to cause infection. Antibiotic irrigation is then followed by dilute betadine irrigation [49]. The wound is then presumed to be sterile [50,51]. Mulcahy and colleagues found they had an 84% success rate with salvage procedures [49,52]. The same principles are applied to a salvage procedure for an AUS. All components are removed, and, after copious surgical site irrigation, a new device is reimplanted [53,54,55]. Ultimately, in the setting of infection, removal of the device, intravenous broad-spectrum antibiotics and delayed reimplantation is the safest course for most patients. A salvage technique is not recommended in cases of sepsis, infection with virulent organisms, extensive cellulitis or necrosis of the tissue, diabetic patients with concomitant ketoacidosis at time of presentation, and a short incubation time from the primary implant [49]. Penile prosthesis salvage was initially motivated by the desire to prevent corporal fibrosis, scarring, and penile shortening, all results of infection on the surrounding tissues. In an infected AUS, these factors are not necessarily at play, but placement of a device following prior device field infection in a delayed fashion is technically challenging due to the significant scar tissue from prior infection.

#### 2.2.2. Urethral Erosion

Another complication that is typically identified later in the postoperative period is urethral erosion. As previously noted, urethral erosion greatly increases the risk of developing device infection, so early identification and intervention is critical. Urethral erosion is more common in patients who have undergone radiation, multiple reconstructive procedures of the urethra, prior device infection, or patients who have a relatively smaller cuff size [48]. There are also data that suggest hypogonadism secondary to either de novo low serum testosterone or ablative therapy for prostate cancer increases the risk of device erosion [15]. Signs and symptoms of urethral erosion include new onset gross hematuria, dysuria, leakage, or urinary tract infection with associated perineal pain. Erosions of the pump are often easily visualized, but diagnosis of urethral cuff erosion requires cystourethroscopic evaluation. Data analysis suggests that urethral erosion occurs in 8% of patients with multiple series reporting erosion rates ranging from 3.3 to 27.8%. Agarwal and colleagues posited three mechanisms for urethral erosion including urethral injury/shallow initial dissection at time of AUS placement, poor quality or ‘high risk’ urethra, and traumatization of the device [7,41]. In a retrospective analysis of the literature, they observed a peak in the first two years following prosthesis implantation and then persistent events up to ten years following implantation. AUS erosion is managed by explantation of the prosthesis. Urinary diversion either by placement of a suprapubic tube or foley catheter per urethra at the time of explantation is standard management. Oftentimes urethroplasty is required later due to the development of scar and stricture at the site of erosion. Stricture formation occurs due to healing by secondary intention at this site. If stricture is encountered following explantation, patients require urethroplasty, thus adding an additional procedure and reoperation in the perineum. This delays the patient desired outcome of continence and results in a more challenging operation due to scar tissue. There are data that suggest either augmented urethroplasty with onlay or inlay at time of cuff erosion reduces the rate of urethral stricture as compared to placement of a urethral catheter or primary anastomotic repair [56,57].

Urethral erosion may also occur in the setting of traumatic catheterizations. This may occur when patients present to care and a healthcare worker attempts catheterization of their bladder while not aware that the patient has an artificial urinary sphincter. Erosion can be an unfortunate outcome as a result of this. This complication may occur when patients present to care unable to provide the necessary information while in urinary retention or following a traumatic accident in which they are obtunded. Although patients are educated that they should inform all providers that they have an implant, existing literature suggests that catheterization of an active urethral sphincter occurs commonly and can result in urethral erosion, device infection, and ultimately explantation of the device. Extensive patient education is necessary in order to prevent this. It has been recommended that patients with artificial urinary sphincters wear medical alert bracelets, not unlike with other implanted prosthetics [18,41].

#### 2.2.3. Device Malfunction and Urethral Atrophy

Device failure secondary to malfunction or urethral atrophy are other common postoperative complications. These outcomes are less complications than outcomes of device wear. Patients who present with recurrent urinary incontinence are those in whom clinicians should suspect device failure or urethral atrophy. Classically, these patients will report progressive, worsening stress urinary incontinence after a period of significant improvement following prosthesis implantation. Evaluation of patients with these symptoms depends heavily on physical examination of the patient and their device, testing how the device cycles, and cystourethroscopic evaluation of the device and the urethra. Management of device failure is guided by which component has failed and the patient’s quality of life. Notably, in a large case series conducted by mail-in response, 51 of 65 respondants who were between 5 and 10 years from surgery reported a quality of life as at least “much better” than their preoperative quality of life [34]. Interestingly, in the cohort of patients greater than ten years out from surgery, 54 of 67 respondents reported similar quality of life improvement [17]. This suggests that those who have function devices are satisfied with the improvements the device made in their continence.

If it can be determined preoperatively, usually just that component can be replaced. However, often it cannot be determined which component has failed and the entire device must be replaced. Some prosthetic urologists advocate for a ‘drain and retain’ approach for the pressure regulating balloon (PRB) [58,59]. In these instances, the entire system is drained of fluid, the cuff and button are removed, and the PRB is left in situ. Retrieval of the PRB often requires extensive adhesiolysis. This not only extends operative time, increasing the risk of infection, but also can lead to inadvertent vascular injury. Cefalu and colleagues reported five years of data regarding their experience with the drain and retain management strategy for both AUS PRBs and IPP reservoirs. They reported no statistical difference in infection, rate of balloon or reservoir hernia, or device malfunction when comparing those who had their PRBs or reservoirs removed with those who had them left in situ. They do acknowledge, as noted below, that these retained devices have the potential to herniate, obstruct, become infected, or erode into various abdominal structures [60]. Yet the authors argue that the benefits of the ‘drain and retain’ approach far outweigh the risks of a retained, defunctionalized device component.

Urethral atrophy is the appearance of a “waisted” or “hourglass” appearance to the tissue that underlies the urethral cuff. There is some debate as to whether this entity exists as an underlying cause of device failure [61,62]. Some researchers have posited urethral atrophy as an event that precedes urethral erosion [41]. Urethral atrophy can be viewed as a type of device malfunction because incontinence occurs due to the urethral cuff no longer providing adequate coaptation due to the loss of tissue. This necessitates, at minimum, replacement of that component either at the location of the previous device, which will often require a smaller size cuff, or at an alternative location at the bulbar or sometimes pendulous urethra. Others have trialed tandem device placement as a means of providing greater control of stress urinary incontinence in the setting of urethral atrophy. This approach is driven by prosthetic surgeon concern for cuff downsizing as the use of a smaller cuff size is associated with an increased risk of erosion but can produce improved continence [63,64].

An approach which attempts to mitigate this risk of erosion is transcorporal placement of the new cuff. This technique has the added benefit of providing additional tissue between the device and the urethra and permits for a larger cuff to be fit to the patient. Notably, transcorporal cuff placement has been observed to have decreased risk of erosion and decreased incidence of explantation specifically in radiated and high-risk urethras. In the same study, erectile function was preserved in 50% of the population studied [26].

#### 2.2.4. Less Common Complications

Though not frequently reported, the less common complications of AUS implantation are critical for prosthetic surgeons to consider in patients who present with symptoms which are not consistent with the complications routinely seen or expected. A case series by Cui and colleagues highlights some of these complications. The series discusses three patients who had undergone prosthetic surgery, either AUS or inflatable penile prosthesis (IPP) implantation, which like the AUS has a reservoir of fluid. They observed complications like erosion of components into pelvic viscera, specifically the cecum [65]. This may occur when a reservoir, meant to be placed in a high submuscular position, is placed within the abdominal cavity. They similarly observed the fluid reservoir of an IPP to be compressing the external iliac vein. This caused lower extremity swelling and deep vein thrombosis. This required extirpation of the previously placed reservoir and placement in an alternative location. This same phenomenon can occur with bowels, as patients have been reported to present with bowel complications secondary to ectopically placed reservoirs [31,66]. Management of these complications requires multispecialty coordination to treat as they are often outside the sphere of urologic expertise.

## 3. Discussion

Awareness of the possible outcomes and complications of AUS implantation aid in the early identification and intervention in patients whose devices are at risk or who may develop significant morbidity and mortality from their complications. It should be noted that many case series and large trials are published by high volume prosthetic urologists. In this way, this gives budding prosthetic urologists and general urologists who desire a practice incorporating prosthetic urology insight into the complications of the most experienced in the field. This expert bias also implies that the published complication rates of highly experienced prosthetic urologists are likely lower than those who do not have the same high-volume practice. Also, due to the nature of prosthetic urology as a field, with a large majority of the AUS implants being performed by a minority of urologists, the best quality data are found within large volume case series and there is a dearth of prospective randomized trials. Limited study sizes and a reporting bias likely impact both the real-world rates of complication, especially in outcomes like infection and urethral erosion. Other limitations in the data available for review include the scarcity of large studies focusing specifically on complications related to AUS implantation and inconsistent language within the literature. For instance, in the available studies for review, postoperative complications are noted as those that occur within thirty days postoperatively, while some authors place the cutoff for postoperative complications as far out as ninety days. Despite these limitations, this review provided information on the most commonly reported complications that arise following AUS implantation. Early recognition of these complications is critical for reducing morbidity and further harm. By outlining these complications, their presentations and their likely outcomes, these authors hope that readers may be able to quickly recognize any complications that may arise in their practice, may that be as the implanter or as a general urologist managing the care of a patient with an implant.

## 4. Future Directions

Moving forward, study of artificial urinary sphincter complications should seek to use a standard timeframe to define perioperative and late postoperative events. Published studies are currently skewed toward the highest volume prosthetic urologists. Studies seeking to understand how non-fellowship trained urologists fare when compared to those in the academic setting may aid in patient counseling and expectation setting for device longevity, rates of infection, and other aforementioned complications. In light of this, compiling data from large case series or retrospective studies may be of use in constructing tools to facilitate patient counseling. These tools could come in the form of risk calculators that take into account patient medical and surgical risk factors to estimate percent chance of erosion, infection, or device failure. Similarly, as the AMS 800^TM^ has dominated the field of male continence surgery over the past three and a half decades, investigation of new and alternative devices is encouraged, as the innovation generated in a competitive market reaps benefits and options for patients and prosthetic surgeons.

## 5. Conclusions

Complications of artificial urinary sphincter placement range from minimally problematic to potentially life-threatening. To our knowledge, this is the first review of all reported complications of AUS surgery. Perioperative complications like scrotal hematomas, urinary tract infection, hematuria, and retention may lead to increased interaction with health systems following surgery but can often be managed with conservative measures. Reoperation in the perioperative setting may be necessary for some large hematomas, pump malposition or migration, and in severe cases, device infection. In the perioperative setting, if the patient is stable without extensive overlying cellulitis, a salvage procedure can be considered to avoid a more difficult two-stage explantation–reimplantation. If erosion is the underlying cause of the patient’s infection, a surgeon experienced in urethroplasty can consider in situ urethroplasty. Device longevity remains a problem in prosthetic urology and is impacted by patient factors like prior history of radiation and the high-risk urethra. 

## Data Availability

The data that support the findings of this study are openly available in the NIH National Library of Medicine at https://pubmed.ncbi.nlm.nih.gov/, accessed on 1 September 2023.
